# Crystal structure of *catena*-poly[[(μ-6-{[bis­(pyridin-2-ylmeth­yl)amino]­meth­yl}pyridine-2-carboxyl­ato)copper(II)] perchlorate aceto­nitrile monosolvate]

**DOI:** 10.1107/S2056989019006285

**Published:** 2019-05-14

**Authors:** Giacomo Cioncoloni, Claire Wilson, Isolda Roger, Mark D. Symes

**Affiliations:** a WestCHEM, School of Chemistry, University of Glasgow, University Avenue, Glasgow, G12 8QQ, United Kingdom

**Keywords:** crystal structure, coordination compound, nitro­gen donor ligands

## Abstract

The crystal structure of the title compound, {[Cu(C_19_H_17_N_4_O_2_)]ClO_4_·C_2_H_3_N}_*n*_, is reported and compared to similar structures in the literature. The compound crystallizes in the monoclinic space group *P*2_1_. The unit cell contains one complex mol­ecule in addition to perchlorate as the counter-ion and solvent (aceto­nitrile). The crystal packing evinces extended chains whereby the carboxyl­ate moiety on the 6-carboxyl­ato-2-(pyridyl­meth­yl)bis­(pyridin-2-ylmeth­yl)amine ligand bridges between two different copper centers in adjacent mol­ecules. This packing arrangement for the title compound appears to be unique when compared to allied structures in the literature. The perchlorate anion showed signs of disorder and its oxygen atoms were modelled over two sets of partially occupied sites, the occupancy of which was competitively refined to 0.564 (12)/0.436 (12). The crystal studied was refined as a two-component inversion twin.

## Abstract 2

The crystal structure of the complex Cu^II^-6-carboxyl­ato-2-(pyridyl­meth­yl)-bis­(pyridin-2-ylmeth­yl)amine is reported and compared to similar structures in the literature. The title compound is observed to form extended chains in the solid state, in contrast to the literature examples discussed.

## Chemical context   

A key part of the natural nitro­gen cycle is the reduction of nitrite to nitric oxide by denitrifying bacteria. The copper-containing nitrite reductases are one of the classes of enzymes that undertake this reduction reaction (Maia & Moura, 2014 ▸). Previous studies have shown that the active site of these enzymes consists of a Cu center coordinated by three N-donor ligands, with the coordination environment being completed by either water or nitrite, depending on the progress of the catalytic cycle (Godden *et al.*, 1991 ▸). This realization has spawned a large number of studies examining the use of copper centers held by multi-dentate N-donor ligands as mimics of the active sites of the copper-containing nitrite reductases (Wasser *et al.*, 2002 ▸; Timmons & Symes, 2015 ▸). In this context, there has been inter­est from ourselves (Cioncoloni *et al.*, 2018 ▸) and others (Komeda *et al.*, 1995 ▸; Nagao *et al.*, 1996 ▸; Orain *et al.*, 2013 ▸) in employing copper complexes based on the tetra­dentate ligand tris­(2-methyl­pyrid­yl)amine (TMPA) as electrocatalysts for the reduction of nitrite to nitric oxide, mimicking some of the activity of the copper nitrite reductases.

In the course of our previous study (Cioncoloni *et al.*, 2018 ▸), we reported two TMPA-based copper complexes that were electrocatalysts for nitrite reduction to NO: [Cu(OH_2_)(TMPA-CO_2_)]^+^ [where one of the TMPA pyridines bears a carboxyl­ate unit that can coordinate to the Cu center and in which the ligand can be named as 6-carboxyl­ato-2-(pyridyl­meth­yl)bis(pyridin-2-ylmeth­yl)amine] and the methyl­ated analogue of this complex, [Cu(OH_2_)(TMPA-CO_2_Me)]^2+^. The acid-bearing complex was found to be the more active of the two electrocatalysts. However, although we were able to report the crystal structure of the methyl ester complex after recrystallization from aceto­nitrile solution, and also the structure of the acid-bearing complex when coordinated to nitrite ([Cu(NO_2_)(TMPA-CO_2_)]), we were not able to obtain similar data for [Cu(TMPA-CO_2_)]^+^ when not bound to nitrite. This was a source of considerable frustration to us at the time, as the structure of the acid-bearing complex prior to nitrite addition constituted a ‘missing link’ in our characterization of this suite of compounds.
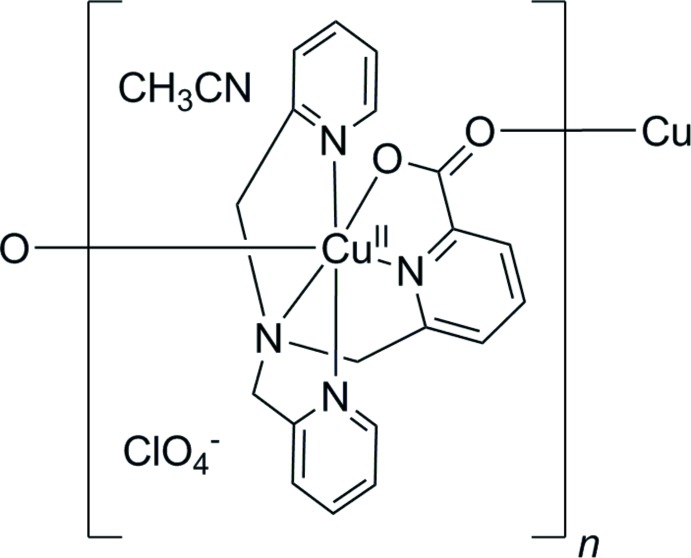


Herein, we report that we have now indeed been able to obtain crystals of the acid-bearing complex in the absence of nitrite ([Cu(TMPA-CO_2_)]^+^) by vapour diffusion of diethyl ether into an aceto­nitrile solution of [Cu(OH_2_)(TMPA-CO_2_)]^+^. In this manuscript, we report this crystal structure and compare it to the structures of [Cu(NO_2_)(TMPA-CO_2_)], [Cu(NCCH_3_)(TMPA-CO_2_Me)]^2+^, and other allied structures from the literature.

## Structural commentary   

**Figure 1:**
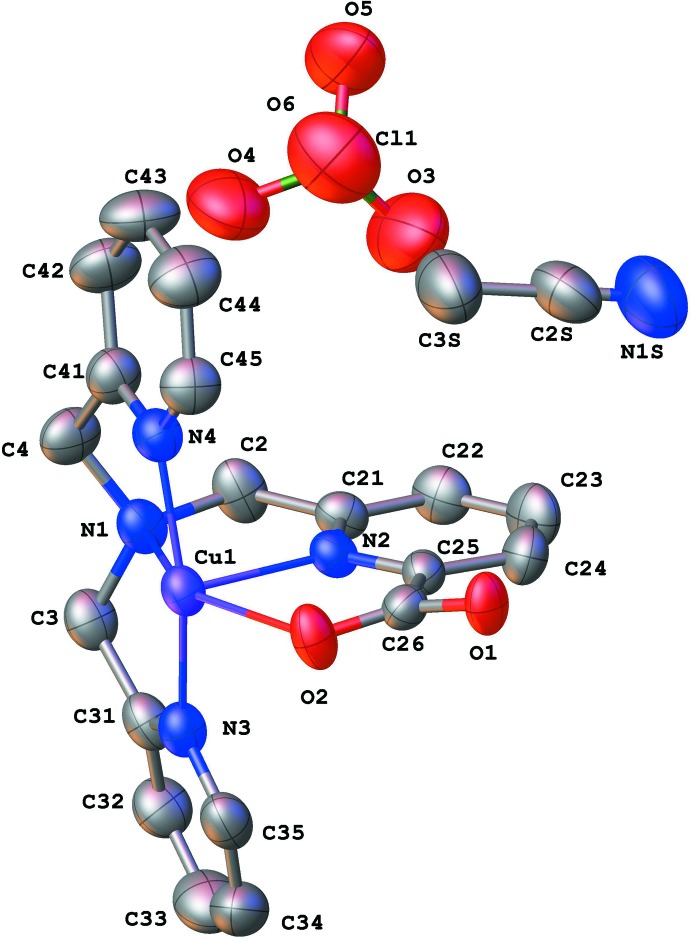
View showing the asymmetric unit and atom labelling. Displacement ellipsoids are drawn at 50% probability level. The minor disorder component of the perchlorate ion and H atoms are omitted for clarity. Colour scheme: C = grey, Cl = green, Cu = purple, N = blue, O = red.

The Cu ion in [Cu(TMPA-CO_2_)]^+^ is in a hexa-coordinated environment where the two oxygen atoms of the carboxyl­ate group are coordinated by two symmetry-related Cu centers, thus forming extended chains (see Figs. 1 ▸ and 2 ▸). This suggests that the carboxyl­ate group is deprotonated. The presence of a perchlorate anion in the unit cell, coupled with the hexa-coordinate geometry, suggests that the copper is in the +2 oxidation state. The geometry around the Cu center is considerably distorted from octa­hedral, with all the vertices showing significant deviations from their positions in the platonic scenario as evinced by the following bond angles: N1—Cu1—O2 = 150.1 (2)°, O1^i^—Cu1—N2 = 161.0 (2)° and N4—Cu1—N3 = 158.7 (2)° [symmetry code: (i) −*x* + 1, *y* − 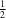, −*z*].

In terms of bond lengths, the Cu—N1 bond length is long [2.296 (6) Å], consistent with N1 being a tertiary amine, rather than a pyridine nitro­gen. The Cu—N_pyrid­yl_ lengths are all significantly shorter than this Cu—N_alk­yl_ inter­action, covering the range 2.000 (5)–2.076 (6) Å. There is an intriguing disparity in the lengths of the two Cu–oxygen inter­actions, with Cu—O1^i^ being very short [1.965 (5) Å] in comparison to the Cu—O2 bond length [2.387 (5) Å]. Coupled with the long Cu—N1 bond, this implies that the N1—Cu1—O2 axis is displaying the Jahn–Teller elongation characteristic of many octa­hedral Cu^II^ complexes. Indeed, this Jahn–Teller effect manifests along the same axis as we observed previously for the related complex [Cu(NO_2_)(TMPA-CO_2_)] (Cioncoloni *et al.*, 2018 ▸). Table 1 ▸ summarizes some selected geometric parameters for [Cu(TMPA-CO_2_)]^+^.

## Supra­molecular features   

The crystal structure shows that the carboxyl­ate moiety on the modified TMPA ligand bridges between two different copper centers, with the result that extended chains of complexes form (see above for a discussion of these coordination bonds). Between the chains, there is some evidence for weak hydrogen bond-type inter­actions between the perchlorate oxygen atoms and the protons on the –CH_2_– units in the TMPA ligands of complexes in neighbouring chains, and/or between the perchlorate anions and protons on the pyridine rings of some of the TMPA ligands (see Table 2 ▸). In this sense, the perchlorate ions can be considered as bridging between adjacent chains of complexes. Meanwhile, the aceto­nitrile mol­ecule forms a hydrogen bond between its nitro­gen and a C—H proton on one of the aromatic rings of the ligand, whilst simultaneously engaging in a hydrogen-bond-like inter­action involving one of its methyl protons and the oxygen on a nearby perchlorate anion. However, there would appear to be no direct supra­molecular inter­actions between adjacent chains.

## Comparison with related structures   

Kojima and co-workers have previously reported complexes of this same (TMPA-CO_2_)^−^ ligand with Ru^III^ and Cr^III^ (Kojima *et al.*, 2010 ▸; Kotani *et al.*, 2015 ▸). These complexes all display a distorted octa­hedral geometry in which the metals coordinate to the three pyridyl nitro­gen atoms and the alkyl nitro­gen in the TMPA backbone as well as to one of the carboxyl­ate oxygen atoms. However, in these examples the sixth coordination site is occupied by either chloride or BF_4_^−^ (which binds through an F atom) and so the complexes do not form extended chains in the solid state (the carboxyl­ate does not bridge between adjacent metal centers). Likewise, Lonnon *et al.* (2003 ▸) have described a Co^III^ complex of this ligand in which the carboxyl­ate coordinates to the metal center and where the sixth ligand is chloride. Again, the presence of this monodentate ligand means that these mol­ecules exist as discrete complexes in the solid state. The metal–O_carboxyl­ate_ distances in the Cr^III^ and Co^III^ complexes reported by Kojima and co-workers and Lonnon *et al.* are not dissimilar to the Cu1—O1 distance we observe [Cr—O = 1.959 Å and Co—O = 1.924 Å compared to Cu1—O1 = 1.965 (5) Å in our case].

In the aforementioned Ru^III^, Cr^III^ and Co^III^ examples, the metal–chloride and metal–BF_4_^−^ inter­actions tend to be long, and the pyridine bearing the carboxyl­ate unit is found to be *trans* to this chloride or BF_4_^−^ ligand (and so it tends to display the shortest metal–N_pyrid­yl_ length in the complex). In our case, however, it is precisely this Cu—N_pyrid­yl_ bond between the Cu atom and the pyridine that bears the carboxyl­ate substituent which is the longest of the three Cu—N_pyrid­yl_ bonds. A similar elongation of the Cu—N_pyrid­yl_ bond for pyridines bearing substituents adjacent to the N-donor has been observed previously by Tanaka and co-workers for the complex [CuCl(TMPA-Me)]^+^, where the TMPA pyridine bearing the methyl group exhibited a significantly longer Cu—N inter­action than that found for the unsubstituted pyridines (2.337 *vs.* 1.99 Å; Nagao *et al.*, 1996 ▸). We also noted an analogous elongation in our previously reported crystal structure of the complex [Cu(NCCH_3_)(TMPA-CO_2_Me)]^2+^ (Cioncoloni *et al.*, 2018 ▸). One possible cause of this bond-elongation effect could be steric crowding brought about by the close proximity of the various substituents to the N-donor atom (Symes & Wilson, 2018 ▸). In support of this hypothesis, long Cu—N inter­actions of a similar nature also manifest in certain Cu^II^–tris­(2-methyl­pyrid­yl)amine complexes (where all the pyridines bear substituents next to the N-donors) reported by Reinaud and co-workers (Izzet *et al.*, 2007 ▸).

Suzuki and co-workers have reported two related Cu^II^ complexes with carboxyl­ate-substituted TMPA-like ligands where the carboxyl­ate group binds to the Cu^II^ centre (Hayashi *et al.*, 2002 ▸; Mizuno *et al.*, 2006 ▸), but for both of these structures the metal is only five-coordinate. The same authors have also published the crystal structure of an allied six-coordinate Ni^II^ complex containing an Ni⋯O_carboxyl­ate_ inter­action (2.084 Å) and where the sixth ligand is water (Shiren *et al.*, 2000 ▸). Again, these complexes appear to exist as discrete ions in the solid state and the formation of extended chains of complexes was not reported.

A number of structures in which Cu^II^ is supported by a TMPA-like ligand (which also bears a carboxyl­ate group which coordinates to Cu), but where the linkage between the pyridine groups in the ligand is a bis­pidine have been described by Comba and co-workers (Comba *et al.*, 2016 ▸, 2018 ▸). In these structures, the metal center adopts a distorted octa­hedral geometry, with bonds to each of the three pyridines, two bonds to the amines in the bis­pidine backbone and a final bond to the carboxyl­ate oxygen. Again, then, the carboxyl­ate does not bridge between two Cu centers in neighbouring complexes and so extended chains are not observed.

## Synthesis and crystallization   

The synthesis and characterization of the complex [Cu(OH_2_)(TMPA-CO_2_)](ClO_4_) have been reported previously (Cioncoloni *et al.*, 2018 ▸). Crystals of [Cu(TMPA-CO_2_)](ClO_4_) were grown by vapor diffusion of diethyl ether into a 14 mL vial containing 2 mg of [Cu(OH_2_)(TMPA-CO_2_)](ClO_4_) dissolved in 2 mL of aceto­nitrile. The 14 mL vial was sealed with a plastic cap, which was pierced by a needle, thus retarding the rate of mixing of the anti­solvent into the aceto­nitrile solution. Crystals suitable for diffraction were obtained after 2–3 weeks.

## Refinement   

Crystal data, data collection and structure refinement details are summarized in Table 3 ▸. The perchlorate anion showed signs of disorder and its oxygen atoms were modelled over two sets of partially occupied sites, the occupancy of which was competitively refined to 0.564 (12)/0.436 (12). Similarity distances restraints were applied to Cl—O bond lengths and O⋯O separations, as well as to the oxygen displacement parameters. Hydrogen atoms were placed in geometrically calculated positions (C—H = 0.93–0.97 Å) and refined as part of a riding model with *U*_iso_(H) values set at 1.2*U*_eq_ of the parent carbon atoms, except the methyl hydrogen atoms of the aceto­nitrile which were refined as a rigid rotor with *U*_iso_(H) set at 1.5*U*_eq_ of the methyl carbon atom. The crystal studied was refined as a two-component inversion twin.

## Supplementary Material

Crystal structure: contains datablock(s) I. DOI: 10.1107/S2056989019006285/zl2754sup1.cif

Structure factors: contains datablock(s) I. DOI: 10.1107/S2056989019006285/zl2754Isup2.hkl

Supporting information file. DOI: 10.1107/S2056989019006285/zl2754Isup3.cdx

CCDC reference: 1913833

Additional supporting information:  crystallographic information; 3D view; checkCIF report

## Figures and Tables

**Figure 2:**
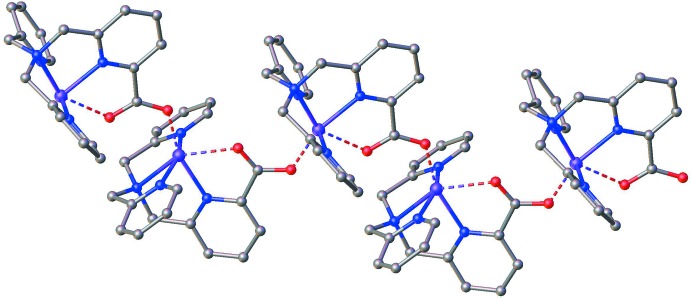
View showing a chain running parallel to the *b* axis generated by the 2_1_ screw axis. Colour scheme: C = grey, Cl = green, Cu = purple, N = blue, O = red.

**Table 1:** Selected geometric parameters (Å, °)

Cu1—O1^i^	1.965 (5)	Cu1—N2	2.076 (6)
Cu1—O2	2.387 (5)	Cu1—N3	2.026 (6)
Cu1—N1	2.296 (6)	Cu1—N4	2.000 (5)
			
O1^i^—Cu1—O2	88.33 (19)	N3—Cu1—O2	99.4 (2)
O1^i^—Cu1—N1	121.6 (2)	N3—Cu1—N1	79.3 (2)
O1^i^—Cu1—N2	161.0 (2)	N3—Cu1—N2	89.5 (2)
O1^i^—Cu1—N3	94.9 (2)	N4—Cu1—O2	101.0 (2)
O1^i^—Cu1—N4	91.7 (2)	N4—Cu1—N1	79.9 (2)
N1—Cu1—O2	150.1 (2)	N4—Cu1—N2	90.8 (2)
N2—Cu1—O2	72.76 (19)	N4—Cu1—N3	158.7 (2)
N2—Cu1—N1	77.3 (2)		

Symmetry code: (i) 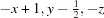.

**Table 2:** Hydrogen-bond geometry (Å, °)

*D*—H⋯*A*	*D*—H	H⋯*A*	*D*⋯*A*	*D*—H⋯*A*
C2—H2*A*⋯O5*A*	0.97	2.21	3.167 (19)	169
C3—H3*A*⋯O3^ii^	0.97	2.54	3.484 (16)	165
C3—H3*B*⋯O2^i^	0.97	2.44	3.149 (10)	130
C22—H22⋯N1*S* ^ii^	0.93	2.53	3.393 (14)	154
C32—H32⋯O5^ii^	0.93	2.49	3.386 (17)	163
C32—H32⋯O3*A* ^ii^	0.93	2.40	3.31 (2)	165
C34—H34⋯O3*A* ^iii^	0.93	2.41	3.23 (2)	148
C42—H42⋯O6^iv^	0.93	2.61	3.43 (2)	147
C42—H42⋯O6*A* ^iv^	0.93	2.64	3.21 (2)	121
C3*S*—H3*SC*⋯O4^v^	0.96	2.54	3.16 (2)	123
C3*S*—H3*SC*⋯O6	0.96	2.46	3.11 (2)	125

Symmetry codes: (i) 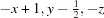; (ii) 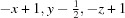; (iii) 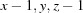; (iv) 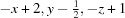; (v) 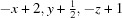.

**Table 3:** Experimental details

Crystal data	
Chemical formula	[Cu(C_19_H_17_N_4_O_2_)]ClO_4_·C_2_H_3_N
*M*_r_	537.41
Crystal system, space group	Monoclinic, *P*2_1_
Temperature (K)	295
*a*, *b*, *c* (Å)	9.320 (3), 9.974 (3), 12.949 (5)
β (°)	109.710 (13)
*V* (Å^3^)	1133.1 (6)
*Z*	2
Radiation type	Mo *K*α
μ (mm^−1^)	1.13
Crystal size (mm)	0.33 × 0.07 × 0.02

Data collection	
Diffractometer	Bruker D8 VENTURE
Absorption correction	Multi-scan (*SADABS*; Bruker, 2016 ▸)
*T*_min_, *T*_max_	0.647, 0.746
No. of measured, independent and observed [*I* > 2σ(*I*)] reflections	15681, 5631, 4168
*R*_int_	0.065
(sin θ/λ)_max_ (Å^−1^)	0.668

Refinement	
*R*[*F* ^2^ > 2σ(*F* ^2^)], *wR*(*F* ^2^), *S*	0.058, 0.147, 1.01
No. of reflections	5631
No. of parameters	346
No. of restraints	203
H-atom treatment	H-atom parameters constrained
Δρ_max_, Δρ_min_ (e Å^−3^)	0.82, −0.36
Absolute structure	Refined as an inversion twin
Absolute structure parameter	0.01 (3)

Computer programs: *APEX3* and, *SAINT* (Bruker, 2016 ▸), *SHELXT2015* (Sheldrick, 2015*a* ▸), *SHELXL2018* (Sheldrick, 2015*b* ▸) and *OLEX2* (Dolomanov *et al.*, 2009 ▸).

## supplementary crystallographic information

### Crystal data

**Table:**

[Cu(C_19_H_17_N_4_O_2_)]ClO_4_·C_2_H_3_N	*F*(000) = 550
*M**_r_* = 537.41	*D*_x_ = 1.575 Mg m^−^^3^
Monoclinic, *P*2_1_	Mo *K*α radiation, λ = 0.71073 Å
*a* = 9.320 (3) Å	Cell parameters from 5520 reflections
*b* = 9.974 (3) Å	θ = 2.3–24.7°
*c* = 12.949 (5) Å	µ = 1.13 mm^−^^1^
β = 109.710 (13)°	*T* = 295 K
*V* = 1133.1 (6) Å^3^	Lath, blue
*Z* = 2	0.33 × 0.07 × 0.02 mm

### Data collection

**Table:**

Bruker D8 VENTURE diffractometer	5631 independent reflections
Radiation source: microfocus sealed tube, INCOATEC Iµs 3.0	4168 reflections with *I* > 2σ(*I*)
Multilayer mirror optics monochromator	*R*_int_ = 0.065
Detector resolution: 7.4074 pixels mm^-1^	θ_max_ = 28.4°, θ_min_ = 2.3°
φ and ω scans	*h* = −11→12
Absorption correction: multi-scan (SADABS; Bruker, 2016)	*k* = −13→13
*T*_min_ = 0.647, *T*_max_ = 0.746	*l* = −17→17
15681 measured reflections	

### Refinement

**Table:**

Refinement on *F*^2^	Secondary atom site location: difference Fourier map
Least-squares matrix: full	Hydrogen site location: inferred from neighbouring sites
*R*[*F*^2^ > 2σ(*F*^2^)] = 0.058	H-atom parameters constrained
*wR*(*F*^2^) = 0.147	*w* = 1/[σ^2^(*F*_o_^2^) + (0.0754*P*)^2^] where *P* = (*F*_o_^2^ + 2*F*_c_^2^)/3
*S* = 1.01	(Δ/σ)_max_ = 0.001
5631 reflections	Δρ_max_ = 0.82 e Å^−^^3^
346 parameters	Δρ_min_ = −0.36 e Å^−^^3^
203 restraints	Absolute structure: Refined as an inversion twin
Primary atom site location: dual	Absolute structure parameter: 0.01 (3)

### Special details

**Table:**

Geometry. All esds (except the esd in the dihedral angle between two l.s. planes) are estimated using the full covariance matrix. The cell esds are taken into account individually in the estimation of esds in distances, angles and torsion angles; correlations between esds in cell parameters are only used when they are defined by crystal symmetry. An approximate (isotropic) treatment of cell esds is used for estimating esds involving l.s. planes.
Refinement. Refined as a two-component inversion twin

### Fractional atomic coordinates and isotropic or equivalent isotropic displacement parameters (Å^2^)

**Table:**

	*x*	*y*	*z*	*U*_iso_*/*U*_eq_	Occ. (<1)
Cu1	0.51948 (8)	0.34337 (8)	0.09197 (6)	0.0371 (2)	
O1	0.4445 (6)	0.7684 (4)	0.0375 (4)	0.0457 (12)	
O2	0.4624 (7)	0.5529 (4)	−0.0021 (4)	0.0496 (12)	
N1	0.5445 (7)	0.2152 (5)	0.2443 (5)	0.0422 (13)	
N2	0.4717 (6)	0.4793 (5)	0.1974 (5)	0.0360 (12)	
N3	0.3055 (7)	0.2687 (5)	0.0532 (5)	0.0417 (13)	
N4	0.7432 (6)	0.3632 (6)	0.1717 (5)	0.0433 (14)	
C2	0.5221 (11)	0.2979 (7)	0.3312 (7)	0.0521 (19)	
H2A	0.616738	0.301036	0.392793	0.062*	
H2B	0.445906	0.255813	0.356277	0.062*	
C3	0.4263 (9)	0.1134 (8)	0.2015 (7)	0.0488 (19)	
H3A	0.401161	0.073697	0.261660	0.059*	
H3B	0.464204	0.042910	0.165958	0.059*	
C4	0.7035 (9)	0.1691 (8)	0.2716 (7)	0.0542 (19)	
H4A	0.707338	0.095062	0.223995	0.065*	
H4B	0.740955	0.137309	0.346750	0.065*	
C21	0.4725 (8)	0.4385 (7)	0.2957 (6)	0.0398 (15)	
C22	0.4344 (9)	0.5259 (8)	0.3657 (7)	0.0501 (18)	
H22	0.437609	0.497017	0.434733	0.060*	
C23	0.3923 (10)	0.6543 (9)	0.3330 (7)	0.057 (2)	
H23	0.363821	0.712700	0.378634	0.069*	
C24	0.3925 (8)	0.6965 (7)	0.2317 (6)	0.0480 (17)	
H24	0.364844	0.783833	0.208113	0.058*	
C25	0.4344 (7)	0.6066 (6)	0.1658 (6)	0.0348 (15)	
C26	0.4491 (7)	0.6439 (6)	0.0574 (6)	0.0376 (14)	
C31	0.2860 (8)	0.1743 (6)	0.1209 (6)	0.0456 (16)	
C32	0.1394 (9)	0.1350 (8)	0.1130 (7)	0.056 (2)	
H32	0.125179	0.073406	0.162637	0.068*	
C33	0.0161 (11)	0.1872 (10)	0.0321 (9)	0.073 (3)	
H33	−0.082139	0.160005	0.025271	0.087*	
C34	0.0397 (10)	0.2809 (9)	−0.0390 (8)	0.065 (2)	
H34	−0.042082	0.316831	−0.095220	0.079*	
C35	0.1858 (9)	0.3197 (7)	−0.0253 (6)	0.0526 (19)	
H35	0.202007	0.383808	−0.072321	0.063*	
C41	0.8035 (9)	0.2817 (7)	0.2577 (6)	0.0506 (18)	
C42	0.9517 (10)	0.2992 (9)	0.3260 (7)	0.066 (2)	
H42	0.991782	0.243111	0.386293	0.079*	
C43	1.0397 (10)	0.3970 (10)	0.3066 (8)	0.072 (3)	
H43	1.140019	0.407794	0.352611	0.087*	
C44	0.9788 (10)	0.4811 (10)	0.2172 (8)	0.070 (3)	
H44	1.036126	0.549964	0.202147	0.084*	
C45	0.8316 (9)	0.4590 (7)	0.1523 (7)	0.0508 (18)	
H45	0.790310	0.513541	0.091217	0.061*	
Cl1	0.8713 (3)	0.4368 (3)	0.6178 (2)	0.0791 (8)	
O3	0.7360 (14)	0.478 (2)	0.6156 (15)	0.122 (5)	0.564 (12)
O4	0.8583 (19)	0.2962 (13)	0.5808 (15)	0.105 (5)	0.564 (12)
O5	0.9871 (17)	0.4452 (18)	0.7185 (10)	0.113 (5)	0.564 (12)
O6	0.933 (2)	0.4989 (17)	0.5431 (13)	0.124 (5)	0.564 (12)
O3A	0.857 (3)	0.3871 (19)	0.7163 (13)	0.118 (6)	0.436 (12)
O4A	0.776 (2)	0.5562 (18)	0.5965 (18)	0.110 (6)	0.436 (12)
O5A	0.820 (3)	0.348 (2)	0.5358 (14)	0.119 (6)	0.436 (12)
O6A	1.0168 (17)	0.481 (2)	0.639 (2)	0.130 (7)	0.436 (12)
N1S	0.6734 (13)	0.8932 (11)	0.4305 (10)	0.103 (3)	
C2S	0.7272 (12)	0.8141 (11)	0.3977 (8)	0.075 (3)	
C3S	0.7983 (13)	0.7027 (12)	0.3537 (11)	0.097 (4)	
H3SA	0.813619	0.731700	0.287459	0.145*	
H3SB	0.732111	0.626035	0.338265	0.145*	
H3SC	0.894629	0.679043	0.407121	0.145*	

### Atomic displacement parameters (Å^2^)

**Table:**

	*U*^11^	*U*^22^	*U*^33^	*U*^12^	*U*^13^	*U*^23^
Cu1	0.0469 (4)	0.0296 (3)	0.0357 (4)	−0.0019 (4)	0.0150 (3)	−0.0008 (4)
O1	0.069 (3)	0.028 (2)	0.044 (3)	0.009 (2)	0.024 (2)	0.008 (2)
O2	0.081 (4)	0.030 (2)	0.042 (3)	−0.001 (2)	0.026 (3)	0.000 (2)
N1	0.057 (3)	0.034 (3)	0.036 (3)	−0.002 (2)	0.016 (3)	0.005 (3)
N2	0.042 (3)	0.033 (3)	0.033 (3)	−0.005 (2)	0.012 (2)	0.001 (2)
N3	0.054 (3)	0.035 (3)	0.039 (3)	0.000 (2)	0.018 (3)	0.002 (2)
N4	0.048 (3)	0.041 (4)	0.043 (3)	−0.002 (3)	0.018 (2)	−0.002 (3)
C2	0.080 (5)	0.042 (4)	0.037 (4)	−0.003 (3)	0.024 (4)	0.006 (3)
C3	0.063 (4)	0.038 (4)	0.050 (5)	−0.006 (3)	0.026 (4)	0.004 (3)
C4	0.055 (4)	0.048 (4)	0.056 (5)	0.003 (3)	0.014 (4)	0.018 (4)
C21	0.045 (3)	0.040 (3)	0.032 (4)	−0.004 (3)	0.011 (3)	0.004 (3)
C22	0.057 (4)	0.061 (5)	0.039 (4)	−0.003 (4)	0.026 (3)	0.001 (4)
C23	0.070 (5)	0.064 (5)	0.049 (5)	0.007 (4)	0.035 (4)	−0.006 (4)
C24	0.052 (4)	0.043 (3)	0.054 (5)	0.009 (3)	0.024 (3)	0.000 (3)
C25	0.034 (3)	0.030 (3)	0.040 (4)	−0.001 (2)	0.012 (3)	−0.002 (3)
C26	0.034 (3)	0.035 (3)	0.041 (4)	0.003 (3)	0.010 (3)	0.002 (3)
C31	0.060 (4)	0.036 (3)	0.045 (4)	−0.009 (3)	0.024 (3)	−0.005 (3)
C32	0.062 (4)	0.047 (4)	0.063 (5)	−0.011 (3)	0.025 (4)	0.003 (4)
C33	0.058 (5)	0.075 (6)	0.090 (8)	−0.016 (4)	0.031 (5)	−0.010 (6)
C34	0.057 (5)	0.058 (4)	0.070 (6)	0.002 (4)	0.006 (4)	−0.007 (4)
C35	0.067 (4)	0.041 (5)	0.045 (4)	0.000 (3)	0.012 (3)	−0.002 (3)
C41	0.056 (4)	0.047 (4)	0.048 (5)	0.003 (3)	0.016 (3)	0.006 (3)
C42	0.056 (5)	0.079 (6)	0.053 (5)	0.005 (4)	0.006 (4)	0.016 (4)
C43	0.047 (4)	0.095 (7)	0.066 (6)	−0.012 (4)	0.006 (4)	0.013 (5)
C44	0.059 (5)	0.076 (6)	0.077 (7)	−0.008 (4)	0.026 (5)	0.013 (5)
C45	0.056 (4)	0.051 (4)	0.047 (4)	−0.004 (3)	0.021 (3)	0.011 (3)
Cl1	0.0638 (13)	0.102 (2)	0.0718 (17)	−0.0176 (13)	0.0237 (12)	−0.0053 (15)
O3	0.087 (9)	0.157 (12)	0.127 (11)	0.012 (9)	0.044 (8)	−0.032 (10)
O4	0.086 (9)	0.103 (10)	0.122 (12)	−0.029 (7)	0.028 (9)	−0.020 (9)
O5	0.114 (10)	0.149 (11)	0.061 (8)	0.027 (9)	0.010 (7)	−0.023 (8)
O6	0.153 (11)	0.136 (10)	0.093 (10)	−0.018 (10)	0.053 (9)	−0.003 (9)
O3A	0.148 (12)	0.125 (12)	0.084 (10)	−0.033 (10)	0.043 (9)	−0.001 (9)
O4A	0.078 (10)	0.135 (13)	0.116 (12)	0.004 (10)	0.033 (9)	0.029 (11)
O5A	0.117 (11)	0.162 (13)	0.067 (10)	−0.009 (12)	0.016 (9)	−0.031 (12)
O6A	0.090 (11)	0.115 (12)	0.167 (15)	−0.025 (10)	0.021 (11)	−0.005 (13)
N1S	0.115 (8)	0.105 (7)	0.099 (8)	−0.010 (6)	0.050 (6)	−0.033 (6)
C2S	0.077 (6)	0.084 (8)	0.061 (6)	−0.018 (5)	0.021 (5)	−0.019 (5)
C3S	0.088 (7)	0.093 (8)	0.112 (10)	−0.006 (6)	0.037 (7)	−0.034 (7)

### Geometric parameters (Å, º)

**Table:**

Cu1—O1^i^	1.965 (5)	C24—C25	1.382 (10)
Cu1—O2	2.387 (5)	C25—C26	1.502 (10)
Cu1—N1	2.296 (6)	C31—C32	1.392 (11)
Cu1—N2	2.076 (6)	C32—H32	0.9300
Cu1—N3	2.026 (6)	C32—C33	1.370 (13)
Cu1—N4	2.000 (5)	C33—H33	0.9300
O1—C26	1.265 (7)	C33—C34	1.381 (14)
O2—C26	1.224 (8)	C34—H34	0.9300
N1—C2	1.466 (9)	C34—C35	1.368 (11)
N1—C3	1.463 (9)	C35—H35	0.9300
N1—C4	1.475 (10)	C41—C42	1.377 (11)
N2—C21	1.334 (9)	C42—H42	0.9300
N2—C25	1.344 (8)	C42—C43	1.352 (12)
N3—C31	1.340 (9)	C43—H43	0.9300
N3—C35	1.330 (9)	C43—C44	1.386 (13)
N4—C41	1.340 (9)	C44—H44	0.9300
N4—C45	1.340 (9)	C44—C45	1.363 (12)
C2—H2A	0.9700	C45—H45	0.9300
C2—H2B	0.9700	Cl1—O3	1.317 (12)
C2—C21	1.499 (10)	Cl1—O4	1.473 (12)
C3—H3A	0.9700	Cl1—O5	1.386 (11)
C3—H3B	0.9700	Cl1—O6	1.422 (12)
C3—C31	1.500 (11)	Cl1—O3A	1.415 (14)
C4—H4A	0.9700	Cl1—O4A	1.456 (14)
C4—H4B	0.9700	Cl1—O5A	1.339 (14)
C4—C41	1.508 (11)	Cl1—O6A	1.362 (13)
C21—C22	1.387 (10)	N1S—C2S	1.095 (13)
C22—H22	0.9300	C2S—C3S	1.501 (16)
C22—C23	1.364 (12)	C3S—H3SA	0.9600
C23—H23	0.9300	C3S—H3SB	0.9600
C23—C24	1.379 (11)	C3S—H3SC	0.9600
C24—H24	0.9300		
			
O1^i^—Cu1—O2	88.33 (19)	C23—C24—H24	120.6
O1^i^—Cu1—N1	121.6 (2)	C23—C24—C25	118.7 (7)
O1^i^—Cu1—N2	161.0 (2)	C25—C24—H24	120.6
O1^i^—Cu1—N3	94.9 (2)	N2—C25—C24	121.5 (7)
O1^i^—Cu1—N4	91.7 (2)	N2—C25—C26	115.0 (6)
N1—Cu1—O2	150.1 (2)	C24—C25—C26	123.4 (6)
N2—Cu1—O2	72.76 (19)	O1—C26—C25	115.2 (6)
N2—Cu1—N1	77.3 (2)	O2—C26—O1	127.1 (7)
N3—Cu1—O2	99.4 (2)	O2—C26—C25	117.7 (6)
N3—Cu1—N1	79.3 (2)	N3—C31—C3	117.5 (7)
N3—Cu1—N2	89.5 (2)	N3—C31—C32	119.8 (7)
N4—Cu1—O2	101.0 (2)	C32—C31—C3	122.7 (7)
N4—Cu1—N1	79.9 (2)	C31—C32—H32	120.1
N4—Cu1—N2	90.8 (2)	C33—C32—C31	119.9 (8)
N4—Cu1—N3	158.7 (2)	C33—C32—H32	120.1
C26—O1—Cu1^ii^	122.9 (5)	C32—C33—H33	120.5
C26—O2—Cu1	112.4 (4)	C32—C33—C34	119.1 (8)
C2—N1—Cu1	110.3 (4)	C34—C33—H33	120.5
C2—N1—C4	112.6 (6)	C33—C34—H34	120.7
C3—N1—Cu1	102.5 (4)	C35—C34—C33	118.7 (8)
C3—N1—C2	113.4 (6)	C35—C34—H34	120.7
C3—N1—C4	116.2 (6)	N3—C35—C34	122.2 (8)
C4—N1—Cu1	100.5 (4)	N3—C35—H35	118.9
C21—N2—Cu1	119.7 (4)	C34—C35—H35	118.9
C21—N2—C25	119.8 (6)	N4—C41—C4	116.6 (7)
C25—N2—Cu1	120.5 (5)	N4—C41—C42	120.4 (7)
C31—N3—Cu1	115.8 (5)	C42—C41—C4	123.0 (7)
C35—N3—Cu1	123.4 (5)	C41—C42—H42	119.6
C35—N3—C31	120.2 (7)	C43—C42—C41	120.8 (8)
C41—N4—Cu1	116.2 (5)	C43—C42—H42	119.6
C45—N4—Cu1	125.0 (5)	C42—C43—H43	120.4
C45—N4—C41	118.4 (6)	C42—C43—C44	119.3 (8)
N1—C2—H2A	108.8	C44—C43—H43	120.4
N1—C2—H2B	108.8	C43—C44—H44	121.3
N1—C2—C21	113.6 (6)	C45—C44—C43	117.4 (8)
H2A—C2—H2B	107.7	C45—C44—H44	121.3
C21—C2—H2A	108.8	N4—C45—C44	123.7 (7)
C21—C2—H2B	108.8	N4—C45—H45	118.1
N1—C3—H3A	109.5	C44—C45—H45	118.1
N1—C3—H3B	109.5	O3—Cl1—O4	108.4 (9)
N1—C3—C31	110.5 (6)	O3—Cl1—O5	116.0 (9)
H3A—C3—H3B	108.1	O3—Cl1—O6	116.9 (11)
C31—C3—H3A	109.5	O5—Cl1—O4	108.4 (9)
C31—C3—H3B	109.5	O5—Cl1—O6	104.4 (9)
N1—C4—H4A	109.5	O6—Cl1—O4	101.6 (9)
N1—C4—H4B	109.5	O3A—Cl1—O4A	102.7 (10)
N1—C4—C41	110.5 (6)	O5A—Cl1—O3A	111.5 (12)
H4A—C4—H4B	108.1	O5A—Cl1—O4A	111.0 (11)
C41—C4—H4A	109.5	O5A—Cl1—O6A	115.6 (13)
C41—C4—H4B	109.5	O6A—Cl1—O3A	108.9 (12)
N2—C21—C2	118.7 (6)	O6A—Cl1—O4A	106.2 (11)
N2—C21—C22	120.8 (6)	N1S—C2S—C3S	178.3 (12)
C22—C21—C2	120.5 (6)	C2S—C3S—H3SA	109.5
C21—C22—H22	120.1	C2S—C3S—H3SB	109.5
C23—C22—C21	119.8 (7)	C2S—C3S—H3SC	109.5
C23—C22—H22	120.1	H3SA—C3S—H3SB	109.5
C22—C23—H23	120.3	H3SA—C3S—H3SC	109.5
C22—C23—C24	119.3 (7)	H3SB—C3S—H3SC	109.5
C24—C23—H23	120.3		
			
Cu1^ii^—O1—C26—O2	−6.7 (10)	C2—C21—C22—C23	178.8 (8)
Cu1^ii^—O1—C26—C25	174.1 (4)	C3—N1—C2—C21	−108.0 (7)
Cu1—O2—C26—O1	167.3 (5)	C3—N1—C4—C41	150.8 (7)
Cu1—O2—C26—C25	−13.6 (7)	C3—C31—C32—C33	−175.1 (8)
Cu1—N1—C2—C21	6.3 (8)	C4—N1—C2—C21	117.6 (7)
Cu1—N1—C3—C31	−38.8 (7)	C4—N1—C3—C31	−147.3 (7)
Cu1—N1—C4—C41	41.2 (7)	C4—C41—C42—C43	176.1 (9)
Cu1—N2—C21—C2	5.6 (8)	C21—N2—C25—C24	−1.7 (9)
Cu1—N2—C21—C22	−177.1 (5)	C21—N2—C25—C26	175.5 (6)
Cu1—N2—C25—C24	175.7 (5)	C21—C22—C23—C24	−1.9 (12)
Cu1—N2—C25—C26	−7.0 (7)	C22—C23—C24—C25	0.5 (12)
Cu1—N3—C31—C3	−12.9 (8)	C23—C24—C25—N2	1.4 (11)
Cu1—N3—C31—C32	168.2 (6)	C23—C24—C25—C26	−175.7 (7)
Cu1—N3—C35—C34	−170.0 (6)	C24—C25—C26—O1	11.0 (9)
Cu1—N4—C41—C4	10.8 (9)	C24—C25—C26—O2	−168.3 (6)
Cu1—N4—C41—C42	−171.7 (7)	C25—N2—C21—C2	−177.0 (6)
Cu1—N4—C45—C44	170.9 (7)	C25—N2—C21—C22	0.3 (9)
N1—C2—C21—N2	−8.0 (10)	C31—N3—C35—C34	1.3 (11)
N1—C2—C21—C22	174.7 (7)	C31—C32—C33—C34	−1.5 (13)
N1—C3—C31—N3	38.2 (9)	C32—C33—C34—C35	−0.9 (13)
N1—C3—C31—C32	−143.0 (7)	C33—C34—C35—N3	1.0 (12)
N1—C4—C41—N4	−39.1 (10)	C35—N3—C31—C3	175.2 (7)
N1—C4—C41—C42	143.5 (8)	C35—N3—C31—C32	−3.7 (10)
N2—C21—C22—C23	1.5 (11)	C41—N4—C45—C44	−2.0 (12)
N2—C25—C26—O1	−166.3 (6)	C41—C42—C43—C44	0.6 (15)
N2—C25—C26—O2	14.5 (9)	C42—C43—C44—C45	−0.7 (15)
N3—C31—C32—C33	3.8 (12)	C43—C44—C45—N4	1.4 (14)
N4—C41—C42—C43	−1.2 (14)	C45—N4—C41—C4	−175.7 (7)
C2—N1—C3—C31	80.0 (8)	C45—N4—C41—C42	1.8 (11)
C2—N1—C4—C41	−76.1 (8)		

Symmetry codes: (i) −*x*+1, *y*−1/2, −*z*; (ii) −*x*+1, *y*+1/2, −*z*.

### Hydrogen-bond geometry (Å, º)

**Table:**

*D*—H···*A*	*D*—H	H···*A*	*D*···*A*	*D*—H···*A*
C2—H2*A*···O5*A*	0.97	2.21	3.167 (19)	169
C3—H3*A*···O3^iii^	0.97	2.54	3.484 (16)	165
C3—H3*B*···O2^i^	0.97	2.44	3.149 (10)	130
C22—H22···N1*S*^iii^	0.93	2.53	3.393 (14)	154
C32—H32···O5^iii^	0.93	2.49	3.386 (17)	163
C32—H32···O3*A*^iii^	0.93	2.40	3.31 (2)	165
C34—H34···O3*A*^iv^	0.93	2.41	3.23 (2)	148
C42—H42···O6^v^	0.93	2.61	3.43 (2)	147
C42—H42···O6*A*^v^	0.93	2.64	3.21 (2)	121
C3*S*—H3*SC*···O4^vi^	0.96	2.54	3.16 (2)	123
C3*S*—H3*SC*···O6	0.96	2.46	3.11 (2)	125

Symmetry codes: (i) −*x*+1, *y*−1/2, −*z*; (iii) −*x*+1, *y*−1/2, −*z*+1; (iv) *x*−1, *y*, *z*−1; (v) −*x*+2, *y*−1/2, −*z*+1; (vi) −*x*+2, *y*+1/2, −*z*+1.
